# Keeping Cool: Use of Air Conditioning by Australians with Multiple Sclerosis

**DOI:** 10.1155/2012/794310

**Published:** 2012-03-28

**Authors:** Michael P. Summers, Rex D. Simmons, George Verikios

**Affiliations:** ^1^MS Australia, 54 Railway Road, Blackburn, VIC 3130, Australia; ^2^The Australian MS Longitudinal Study (AMSLS), Canberra Hospital, P.O. Box 11, Woden, ACT 2606, Australia; ^3^Centre of Policy Studies, Monash University, Building 11E Clayton, IC 3800, Australia

## Abstract

Despite the known difficulties many people with MS have with high ambient temperatures, there are no reported studies of air conditioning use and MS. This study systematically examined air conditioner use by Australians with MS. A short survey was sent to all participants in the Australian MS Longitudinal Study cohort with a response rate of 76% (n = 2,385). Questions included hours of air-conditioner use, areas cooled, type and age of equipment, and the personal effects of overheating. Air conditioners were used by 81.9% of respondents, with an additional 9.6% who could not afford an air conditioner. Regional and seasonal variation in air conditioning use was reported, with a national annual mean of 1,557 hours running time. 90.7% reported negative effects from overheating including increased fatigue, an increase in other MS symptoms, reduced household and social activities, and reduced work capacity. Households that include people with MS spend between 4 and 12 times more on keeping cool than average Australian households.

## 1. Introduction

Heat intolerance has long been known to be a significant issue for people with MS, where an increase in core body temperature is associated with increased neurological symptoms [1–6]. People with MS rate high temperatures as one of the top three factors adversely affecting their symptoms, with stress and insufficient sleep being the other two [2]. Indeed, heat intolerance has a significant impact on the economic situation and quality of life of people with MS and their families [7], and managing heat-related problems is important to maintaining employment of people with MS [8]. While neurological symptoms are frequently exacerbated by heat, these generally return to baseline when core temperature returns to normal, and colder temperatures usually reduce MS symptoms [9–11].

While it is clear that heat frequently exacerbates MS symptoms, patterns of relapse and exacerbation in relation to seasonal climate variations are variable and confounded by other factors such as viral infections and the modifying behaviours of people with MS, who routinely try to minimise their exposure to heat on hot days and nights [12–14]. In Australia, where the main climate variations range from temperate to tropical, residential air conditioning is widely used by people with MS to minimise the impact of hot days and nights on their symptoms. Australian electricity prices are rising rapidly with annual increases of more than 10% annually between 2007 and 2010 [15]. Thus, in addition to the other economic difficulties faced by people with MS [16, 17], affordability of residential cooling is a major issue for many, frequently compounded by an early exit from the labour force and loss of income [18]. In light of the above, *MS Australia *perceived a potential need for government-funded financial assistance for residential electricity costs Australians with MS on low incomes and sought relevant evidence on this issue to determine whether assistance was required and to underpin public policy advocacy and government lobbying efforts if government assistance was warranted. An extensive search of peer-reviewed literature and other sources indicated no studies of air-conditioning use by people with MS. We therefore set out to investigate the home cooling needs of Australians with MS.

## 2. Materials and Methods

### 2.1. Survey

The Keeping Cool Survey consisted of nine questions covering the following topics (see the Supplementary Material available online at doi:10.1155/2012/794310): home air-conditioner use/nonuse and reasons why; ambient temperature range when air conditioning is switched on; age and type of air conditioner (to estimate efficiency); rooms of house that are air conditioned; number of hours the air conditioner is used in particular months; other home modifications to assist cooling; the following question.

“As a person with MS, what happens when you get too hot?” (Tick all that apply).

- Nothing, I cope just fine.
- I lack energy and require more rest.
- Apart from fatigue, my other symptoms of MS become worse.
- I am unable to participate in normal social activities (time with family or friends).
- I am unable to do my normal household duties (e.g., cleaning, cooking, etc.).
- I am unable to work effectively.
- I am unable to look after myself in the usual manner.
- I need more medication to cope.
- I have felt sufficiently unwell to require a doctor or other health professional.
- I have been hospitalised because of heat.

Demographic data for survey respondents was available from the Australian MS Longitudinal Study (AMSLS) database, including sex, age, and postcode. Of the respondents to the Keeping Cool Survey, 66% (1,578) had also participated in one or both AMSLS Economic Surveys conducted in 2003 and 2007. This, along with links to basic demographic information collected in other parts of the longitudinal survey, enabled additional analyses to be undertaken (see Section 2.3 below).

### 2.2. Survey Participants

The AMSLS is a nationwide, longitudinal cohort study described elsewhere [18, 19]. Briefly, the AMSLS maintains a large sample of volunteer participants from all Australian states and territories; 96% of whom have Definite MS by the McDonald diagnostic criteria, according to their neurologist or treating physician. The AMSLS project is approved by the ACT Health Human Research Ethics Committee, an independent National Health and Medical Research Council-constituted human research ethics committee conforming to the ethical standards for human studies as per the 1964 Declaration of Helsinki. The survey was sent to 3,150 consenting participants of the AMSLS in September 2008, of whom 2,385 responded (76%).

### 2.3. Data Analysis

Analyses were undertaken comparing responses on all survey questions between those who would likely be eligible for government-funded assistance and those that would not. Nonparametric tests were used to test categorical variables (Chi-square) and ordinal data (Kruskal-Wallis and Mann-Whitney). No *P* values approached significance, so all subsequent analysis considered the survey population as a whole. Also, there were only three survey participants from the Northern Territory; consequently their data were excluded from the analysis.

Economic modelling for estimating costs of air conditioner use by people with MS was undertaken utilising the survey results. This was necessary as it was found in piloting early versions of the questionnaire that it was not possible to get meaningful responses to direct questions about actual running costs. More details on the economic modelling and associated sensitivity analysis are available elsewhere [20].

Climate data was sourced from the Australian Bureau of Meteorology (BoM). Additionally, the BoM created a new data set combining annual average maximum air temperatures and humidity into a single measure of “apparent temperature” (a commonly used biometeorological measure), for this study.

## 3. Results

### 3.1. Climate

Air-conditioner use at home by people with MS is a direct response to day-to-day weather conditions. One of the difficulties of examining climatic impacts on the use of air conditioners by people with MS is the climatic variation across time and place. Additionally, air temperature data has significant limitations because moderate to high levels of humidity, coupled with hot days and nights, make it more difficult for people to keep cool.

National climate data provides an overview of Australian conditions. The BoM routinely reports data on the number of hot and very hot days and nights, and national annual means for these are reported below in Table 1 [21].

 Australia has experienced an increased number of hot days and nights over time (Table 1), which impacts on the air-conditioning needs of Australians with MS and their associated costs. Temperatures in 2008 when the survey was conducted were typical of the decade at 48.8 days ≥35°C and 80.5 nights ≥20°C. Importantly, 35°C is very hot for people with MS, as the survey results indicated that 29.9°C is the mean external temperature at which Australians with MS turn on their air conditioners. Moreover, there are many more days over 30°C than over 35°C in most areas; for example, across five weather stations in the Sydney area there were 4.4 times more days ≥30°C than there were ≥35°C [21]. 

Table 2 presents the average maximum *apparent temperature* (AT) data for Australian states/territories and capital cities. AT is an adjustment made to the ambient air temperature based on the level of humidity, which also impacts on personal comfort levels. The BoM adjustments use absolute humidity with a dewpoint of 14°C as the reference point (with slight adjustments depending on the temperature). If the humidity is higher than the reference point, then the AT is higher than the air temperature, and if the humidity is lower than the reference point, then the AT is lower than the air temperature.

In Table 2, AT is ordered from highest to lowest, and ATs for capital cities are included along with data for the states and territories within which AT can vary by as much as 16°C. Australia's population distribution is highly skewed towards major urban centres; hence, the AT for the capital cities may better represent the actual impacts on a state and territory populations.

### 3.2. Survey

The demographic profile of survey participants was 79.0% female, and the mean age of the participants was 52 years, with a range of 25–83 years. Also for the 1,578 participants for whom economic data was available, 32.2% were likely to be eligible for a government-funded rebate on their electricity bills if a medical cooling rebate was in place.

Air conditioners were used by 81.9% of survey participants, with an additional 9.6% stating that they could not afford an air conditioner. In comparison, 66.4% of households nationally had an air conditioner in 2008 [22].

Responses to the question “What happens when you get too hot?” are summarised below in Table 3. Only 10.3% of respondents stated that they coped well in the heat and had no problems as a consequence of getting too hot. The most common issue was fatigue at 83.7%, followed by increases in symptoms other than fatigue at 61.9%. Just under half of the respondents also reported reduced capacity for social, household and work activities, and self-care. Small but notable proportions of people with MS also reported impacts on their use of medication (9.1%), doctor or other health professional visits (7.4%), and hospitalisation (3.4%). Totals exceed 100% as categories were not mutually exclusive.

The mean national average external air temperature at which people turned on their air conditioners was 29.9°C. Tasmania was the state with the lowest mean at 26.4°C, with the highest in South Australia and Western Australia at 30.7°C and 30.2°C, respectively.

**Figure 1:**
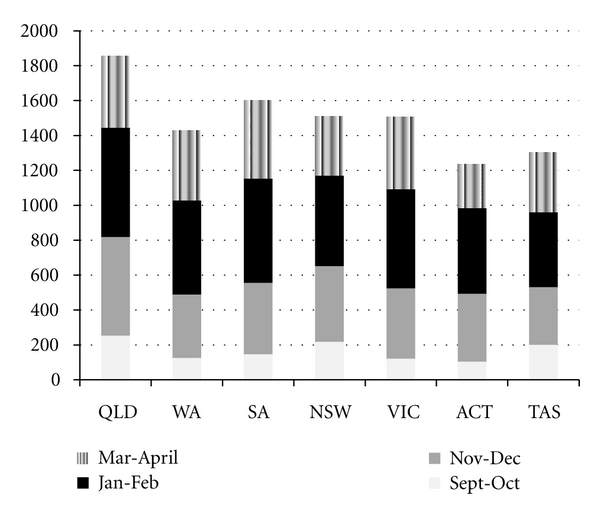
Mean hours of air-conditioner use per household.

Figure 1 describes the imputed mean total hours of air-conditioner use per household across states and territories from September to April. Nationally the mean was 1,557 hours, with variations across states and territories. Imputed means were derived by using the midpoints from the categories selected by survey respondents: 0 = 0; 1–6 hrs = 3.5 hrs; 7–12 hrs = 9.5 hrs; 13–18 hrs = 15.5; 19–24 hrs = 21.5.

The types of air conditioners (evaporative or heat exchanger) used were almost identical to that of the general population [23], with heat exchangers dominating (ranging from 60 to 98%) and higher levels of evaporative more common in the driest areas (Western Australia, South Australia, Victoria, and Australian Capital Territory 30–40%). Newer air conditioners (0–3 years old) were used in 36.2% of households, with 4–9-year-old units at 42.8% and those 10 years old or more at 20.9%. When the survey was conducted, inverter technology, which increases heat exchanger air conditioner efficiency by 25–35%, was only present in those units 0–3 years old. Nationally, 34.1% were cooling one room only, 32.7% were cooling two rooms, and 33.1% were cooling 4 rooms or more (none stated that they were cooling 3 rooms).

Finally, respondents were also asked about their use of various measures to increase the thermal performance of their homes. Nationally, 40.0% used external window coverings such as blinds and awnings, 80.5% used internal window coverings, 69.8% had ceiling insulation, 18.9% had roof vents, and 26.6% had wall insulation. While the figures for insulation are generally low by North American and European standards, MS households exceed national means for both wall (19%) and ceiling (59%) insulation [21].

### 3.3. Costs

Economic modelling was undertaken to convert the survey results into cost data (see Section 2.3 above). Four different sets of costs have been calculated based on different prices for electricity. The first two sets, based on $0.15/kWh and $0.20/kWh, represent the range of prices across Australia when the survey was conducted in 2008. The other two sets of prices, $0.25 and $0.30, represent the range of prices in 2011.

The economic modelling results are in Table 4. For comparison, estimated nonevaporative air-conditioner cost data for households across Australia in 2007 are included under the heading “All” [23]. All figures are in Australian currency.

## 4. Discussion

The present study has provided strong evidence that use of residential air conditioning by Australians with MS is a major and common strategy for minimising the impact of a warm to hot climate. It also appears that people with MS in Australia report a much higher incidence of heat sensitivity than those living in cooler climates. A recent survey in Sweden found that only 58% reported heat sensitivity [4], compared to almost 90% in Australia. This difference would appear to derive from climatic differences where Australians are exposed to much higher temperatures (see Tables 1 and 2) consistently compared to their Swedish counterparts where the temperature rarely exceeds 25°C at the peak of summer. Other research across a variety of locales has typically found a range of 60–80% of people with MS self-reporting as heat sensitive in relation to symptoms increasing with exposure to heat [5]. This further emphasises the importance of air conditioning for people with MS to keep cool in warmer climates such as Australia.

Australian households that include people with MS are more likely than households nationally to (a) have an air conditioner, (b) use the air conditioner more often, and (c) incur the concomitantly higher electricity bills. These data are fundamental to the argument that government-funded financial assistance should be available to those on low incomes requiring medical-related cooling in the form of residential air conditioning. Moreover, our results show that MS households had taken additional steps relative to other households to improve the thermal efficiency of their homes, above and beyond any government-funded contribution. The survey has therefore provided information for policy makers about the considerable problems people with MS incur with warm to hot weather, including regional variation in usage and need.

Nationally, people with MS spend between 4 and 12 times more on running their air conditioners than other households, with a national average of about 4 times more (excluding evaporative air coolers). Little research has been done to track household use of air conditioners generally, but one comparable survey (based on recall and the completion of a questionnaire similar to the Keeping Cool Survey) performed in Victoria in 2008 found that the average total hours of air conditioner use was 107 hours [24], compared to the present survey results in Victoria of 1,508 hours. This indicates that MS households in Victoria run their air conditioners by 14 times more hours annually than other households. For many households these additional costs must be met from relatively low incomes, with relatively high unemployment associated with MS [18].

The high number of hours that MS households use their air conditioners relative to other households probably arises from several factors. People with MS are more likely to be at home as many are not working in paid employment, and they are more likely to be indoors during hot weather to escape the heat. The outside air temperature at which they turn on their air conditioners may also be at a lower threshold than other households given their heat sensitivity. While some of this higher use is probably slightly reduced through the higher levels of wall and ceiling insulation in MS households, these differences in thermal efficiency are relatively minor.

Strengths of the present study include the large national sample size (almost 2,400 households with people with MS) and the ability to subanalyse use of air conditioning by climatic and political regions. The latter enabled collation of data relevant to government policy makers in different Australian states, leading to significant local outcomes in electricity subsidies for people with MS. (Details of such outcomes can be obtained by contacting the corresponding author.) Limitations of the study include the use of retrospective questions on air-conditioning use, which may have introduced some recall bias, though in which direction remains unclear. The inability of households to identify the actual costs of running their air conditioners also necessitated economic modelling to obtain cost estimates.

The sampling for the air-conditioning survey had some biases that are likely to have impacted on the results. Previous examinations of the representativeness of participants in the AMSLS have found that the demographic characteristics of participants are similar to those of other recent studies, supporting the claim that the survey is broadly representative of Australians with MS. Nevertheless, there is likely an under-representation of those at the youngest and oldest ends of the age distribution and perhaps some bias towards over-representation of females [18].

## 5. Conclusions

In Australia people with MS use air conditioners extensively to keep cool at home when ambient external temperatures reach levels that begin to exacerbate their symptoms. The economic costs of this are increasingly problematic for low-income households, and the research results have been essential in demonstrating the need for government assistance to these households. Subsequent campaigns by MS Australia to obtain medical cooling rebates from several state governments have been successful, in part because of the presentation of such hard data to government policy makers. Therefore, the use of patient self-reported data on salient life issues, facilitated by the existence of well-characterised national cohorts like the Australian MS Longitudinal Study, can effectively be used for government lobbying to assist people with MS [25].

Finally, high levels of air-conditioner use by people with MS, and the need for assistance with electricity bills, are not the only policy implications of the survey. Policy advocacy work also needs to be undertaken in relation to the replacement of old and inefficient air conditioners with more efficient units that have the capacity to cut operating costs substantially (up to 50–65%). Also, given that peak demand for electricity in Australia is during the afternoon and early evening of hot days, this is also when electricity supply overloads and blackouts are most likely to occur, making it essential for people with MS to have plans in place to minimise their exposure to heat when electricity is not available to their homes.

## Supplementary Material

‘A copy of the original *Keeping Cool Survey* that was used in this research is presented below.'

## Figures and Tables

**Table 1:** Hot and nights days in Australia over time.

	1957–2009 Mean	2000–2009 Mean
Days ≥ 35°C	43.8	52.3
Days ≥ 40°C	12.0	15.0
Nights ≥ 20°C	69.6	79.5
Nights ≥ 25°C	23.5	26.6

**Table 2:** Australian mean annual maximum apparent temperature.

State/territory	State/territory apparent temperature **(**Celsius)	Capital city apparent temperature (Celsius)
Northern Territory	31.2°	35.0°
Queensland	30.2°	27.0°
Western Australia	28.0°	24.1°
South Australia	25.0°	21.4°
New South Wales	23.1°	22.9°
Victoria	19.5°	19.7°
Australian Capital Territory	16.4°	18.9°
Tasmania	15.8°	16.5°

Data was compiled for MS Australia by the Bureau of Meteorology in 2008 using 30 year averages from 1976 to 2005.

**Table 3:** What happens when you get too hot?

Percent	*n*	Consequence
10.3	243	Nothing
83.7	1980	Fatigue
61.9	1465	Other symptoms increase
45.4	1074	Reduced social activities
48.8	1154	Reduced household activities
47.1	1114	Reduced work capacity
19.6	464	Unable to look after myself as usual
9.1	216	More medication required
7.4	176	Require a doctor or other health professional
3.4	80	Have been hospitalised because of heat

**Table 4:** Estimated annual mean costs of air-conditioner use in MS and all households.

State	Cost at $0.15/kWh		Cost at $0.20/kWh		Cost at $0.25/kWh		Cost at $0.30/kWh	
	$		$		$		$	
	MS	All	MS	All	MS	All	MS	All
QLD	823	193	1097	258	1371	322	1645	387
WA	580	126	774	168	967	210	1161	252
SA	583	134	778	179	972	223	1167	268
NSW	522	106	696	141	871	176	1045	212
VIC	439	36	585	47	731	59	878	71
ACT	296	50	395	66	493	83	592	100
TAS	292	0	390	0	487	0	585	0
Australia	520	117	693	156	867	195	1040	234
