# Supplementary material for: Keeping Cool: Use of Air Conditioning by Australians with Multiple Sclerosis
Source: Mult Scler Int. 2012 Mar 28;2012:794310. doi: 10.1155/2012/794310 (PMC3323861; doi:10.1155/2012/794310)
Supplement: Supplementary file 1 — ‘A copy of the original Keeping Cool Survey that was used in this research is presented below.' [file 794310.f1.pdf]

Australian MS Longitudinal Study (AMSLS)

"Keeping Cool" Short Survey 2008

Your Study ID Number is: ☐☐☐☐

<u>Purpose of Survey</u>: People with MS sometimes have trouble with hot weather and keeping cool. *MS Australia* would like to know the extent of this problem, so they can work toward improving the situation for people with MS, including making informed requests to government agencies for increased financial assistance for air-conditioning costs, etc. By completing this survey and returning it in the enclosed reply-paid envelope, you will be helping with this advocacy work. Please note there is no guarantee that any survey participant will personally benefit.

Please do not write your name on the questionnaire. Those entering the data will not know who you are, and only anonymous, grouped results will be analysed and made public. Please answer all questions on both sides of the page by <u>ticking the appropriate boxes</u>.

1. Do you use an <u>air conditioner</u> at home to keep cool on hot days or nights?

YES [ ] -- Please go to Question 3. NO [ ] -- Please go to Question 2.

2. I do <u>not</u> use an air conditioner at home because… (please tick all applicable reasons):

(a) I do not have a problem with hot weather [ ]

(b) I do have a problem with hot weather but cannot afford to buy an air conditioner [ ]

(c) I have an air conditioner and need to use it, but cannot afford the electricity [ ]

(d) I have an air conditioner and need to use it, but it is broken [ ]

IF YOU HAVE ANSWERED QUESTION 2, PLEASE GO DIRECTLY TO QUESTION 9, OVER PAGE.

3. How hot is it outside when you when you usually turn your air conditioner on?

20 – 24° C [ ] 25 – 29° C [ ] 30 – 34° C [ ] 35 – 39° C [ ]

4. What type of air conditioner(s) do you have?

Split system [ ] Window unit(s) [ ] Ducted [ ] Evaporative [ ]

5. How OLD is your air conditioner?

0 – 3 Years [ ] 4 – 9 Years [ ] 10 or more Years [ ] Don't know [ ]

6. **Which rooms in the house do you usually try to keep cool with your air conditioner(s)?**

   My Bedroom [ ]   Lounge/Sitting Room [ ]   Kitchen [ ]   Other room(s) [ ]

7. **How many <u>hours</u> do you run your air conditioner each day/night (24-hour period) during the following months of the year:**

   (a) Jan to Feb: 0 hrs [ ] 1 – 6 hrs [ ] 7 – 12 hrs [ ] 13 – 18 hrs [ ] 19 – 24 hrs [ ]

   (b) Mar to Apr: 0 hrs [ ] 1 – 6 hrs [ ] 7 – 12 hrs [ ] 13 – 18 hrs [ ] 19 – 24 hrs [ ]

   (c) May to June: 0 hrs [ ] 1 – 6 hrs [ ] 7 – 12 hrs [ ] 13 – 18 hrs [ ] 19 – 24 hrs [ ]

   (d) July to Aug: 0 hrs [ ] 1 – 6 hrs [ ] 7 – 12 hrs [ ] 13 – 18 hrs [ ] 19 – 24 hrs [ ]

   (e) Sept to Oct: 0 hrs [ ] 1 – 6 hrs [ ] 7 – 12 hrs [ ] 13 – 18 hrs [ ] 19 – 24 hrs [ ]

   (f) Nov to Dec: 0 hrs [ ] 1 – 6 hrs [ ] 7 – 12 hrs [ ] 13 – 18 hrs [ ] 19 – 24 hrs [ ]

8. **In addition to an air conditioner, do you have any other home modifications that help you to keep cool? Please tick any items that apply:**

   <u>External</u> window blinds, awnings or other coverings [ ]

   <u>Internal</u> window blinds, drapes or other coverings [ ]

   Roof insulation [ ]   Roof vents [ ]   Wall insulation [ ]

9. **As a person with MS, what happens to you when you get too hot? (Tick all that apply):**

   (a) Nothing, I cope just fine [ ]

   (b) I lack energy and require more rest [ ]

   (c) Apart from fatigue, my other symptoms of MS become worse [ ]

   (d) I am unable to participate in normal social activities (time with family or friends) [ ]

   (e) I am unable to do my normal household duties (e.g. cleaning, cooking, etc.) [ ]

   (f) I am unable to work effectively [ ]

   (g) I am unable to look after myself in the usual manner [ ]

   (h) I need more medication to cope [ ]

   (i) I have felt sufficiently unwell to require a doctor or other health professional [ ]

   (j) I have been hospitalised because of heat [ ]

END OF SURVEY. Thank you for your participation. Please return this form promptly in the enclosed reply-paid envelope, or mail to:

Australian MS Longitudinal Study, Level 2 East, Building 5, Canberra Hospital WODEN ACT 2606
